# Development of Age-Related Macular Degeneration During Treatment for Diabetic Macular Edema: A Case Report

**DOI:** 10.7759/cureus.77606

**Published:** 2025-01-18

**Authors:** Chiyori Hirai, Emi Shiraishi, Hikaru Tsuchida, Kei Shinoda

**Affiliations:** 1 Ophthalmology, Kikkoman General Hospital, Noda City, JPN; 2 Ophthalmology, Saitama Medical University, Iruma-gun, JPN

**Keywords:** age-related macular degeneration, bilateral involvement, diabetic macular edema, epiretinal membrane, vitrectomy

## Abstract

We report a rare case of a patient who developed age-related macular degeneration (AMD) in one eye during treatment of diabetic macular edema (DME) in both eyes.

A 70-year-old man was followed for diabetic retinopathy for 23 years. At his initial visit in 200X, his decimal visual acuity was 1.0 in both eyes. He underwent panretinal photocoagulation for pre-proliferative diabetic retinopathy (PPDR) in both eyes. Optical coherence tomography (OCT), which was done nine years after the initial visit, revealed a small retinal pigment epithelial detachment (PED) in the left eye. Sixteen years after the initial visit, he developed DME in his right eye and an epiretinal membrane (ERM) in his left eye. Seventeen years after the initial visit, he developed DME in his left eye, and he was treated with sub-tenon triamcinolone acetonide (STTA) injections in the right eye. The STTA was not effective, and we switched to anti-vascular endothelial growth factor (anti-VEGF) injections for both eyes. The anti-VEGF injections were performed 22 times (aflibercept, IVA 12 times; faricimab, IVF 10 times) in the right eye for the DME. His decimal visual acuity fluctuated between 0.2 and 0.9 during this period. The left eye underwent pars plana vitrectomy (PPV) for the DME and ERM 17 years after the initial visit. Although the macular edema was resolved, subretinal fluid and macular neovascularization (MNV) appeared 22 years after the initial visit. Since then, IVF has been performed 10 times, and the decimal visual acuity has improved from 0.4 to 0.8. He underwent bilateral cataract surgery 17 years after the initial visit. Twenty-two years after the initial visit, the left eye developed MNV, located near the PED that had been seen on the OCT image taken 13 years earlier. The relationships of the ERM, DME, and PPV with MNV were not determined. At present, both eyes are responding well to the IVF treatments.

This case will remind clinicians of the possibility of the two diseases coexisting and will lead to a better understanding of the pathogenic mechanisms of both diseases.

## Introduction

Both age-related macular degeneration (AMD) and diabetic macular edema (DME) are progressive retinal disorders, and they can cause severe visual impairments in adults [1,2]. The background factors associated with these disorders include aging and arteriosclerosis, and their pathogenesis is associated with inflammatory factors such as vascular endothelial growth factor (VEGF) and oxidative stress [3-8]. Thus, it is not difficult to imagine that both diseases may occur in the same patient. Epidemiological evidence has shown that there are a certain number of overlapping cases of the two diseases [9,10]. Epidemiological information does not provide information on the relative locations of the lesions between the two diseases, the time of onset, or the course of the disease, including treatment. However, to the best of our knowledge, there are no reports of cases in which both disorders developed in fellow eyes. We report our findings in a rare case in which AMD developed in the fellow eye of a patient who was being treated with intravitreal injections of anti-VEGF in one eye for DME.

## Case presentation

**Figure 1:**
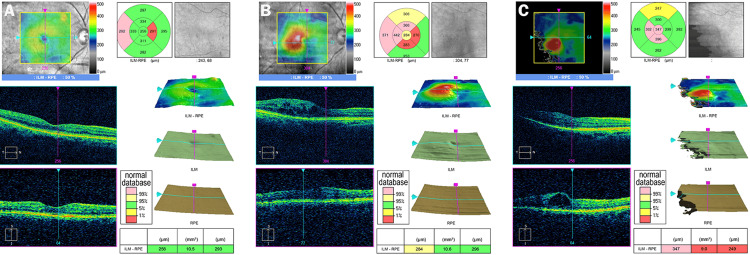
Optical coherence tomographic (OCT) images showing changes over time in the macula of the right eye A. OCT images of the right eye before the onset of macular edema. Visual acuity is 1.0. B. OCT images of the right eye at the onset of macular edema (sixteen years after the initial visit). Macular edema in the central area can be observed. The retinal pigment epithelium (RPE) is irregular (red arrow). Decimal visual acuity is 0.8. C. OCT images of the right eye during the treatment for macular edema (22 years after the initial visit). As in Figure 1B, macular edema in the central area can be observed. Visual acuity is 0.6.

**Figure 2:**
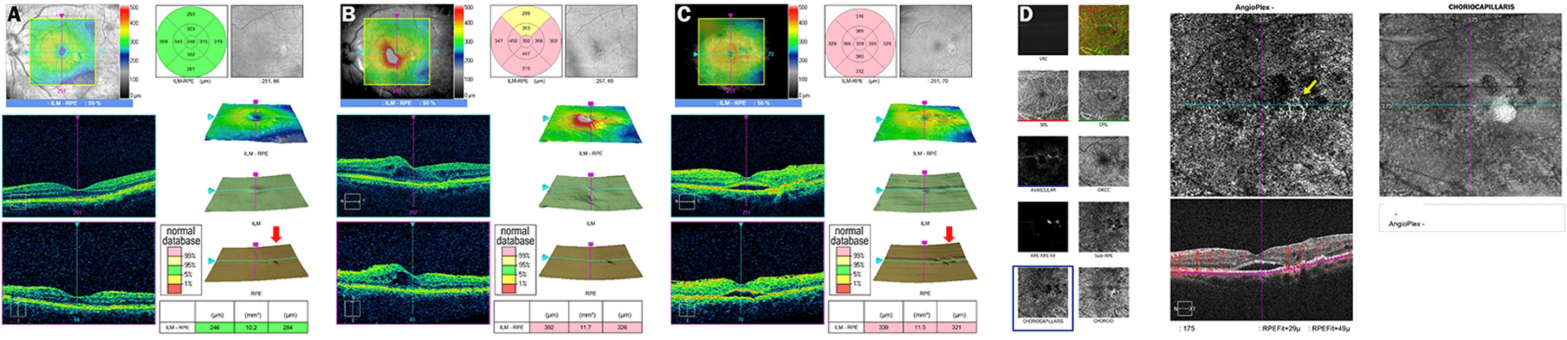
Optical coherence tomographic (OCT) and optical coherence tomography angiographic (OCTA) images showing changes over time in the macula of the left eye A. OCT images of the left eye before the onset of macular edema, on the same day as Figure 1A. Visual acuity is 1.0. B. OCT images of the left eye at the onset of macular edema (17 years after the initial visit). Macular edema in the central area can be observed. The visual acuity is 0.8. C. OCT images of the left eye at the onset of macular neovascularization (22 years after the initial visit). Subretinal fluid and pigment epithelium detachment (red arrow) can be seen in the same position as in the image of Figure 2A. Visual acuity is 0.4. D. OCTA images on the same day as Figure 2C. Macular neovascularization is observed (yellow arrow).

A 70-year-old man has been followed for diabetic retinopathy for 23 years. The clinical course of both eyes and the pathological changes in the macula determined by optical coherence tomography (OCT) and OCT angiography (OCTA) are shown in Figures 1, 2.

At the initial visit in 200X, his decimal best-corrected visual acuity (BCVA) was 1.0 in both eyes, and he underwent panretinal photocoagulation in both eyes for pre-proliferative diabetic retinopathy. The OCT images that were taken nine years after the initial visit revealed a small retinal pigment epithelial detachment (PED) in the left eye (Figure 2A). Sixteen years later, he developed diabetic macular edema (DME) in the right eye (Figure 1B) and an epiretinal membrane (ERM) in the left eye. Seventeen years later, he developed DME in the left eye (Figure 2B), and his BCVA decreased to 0.8 in the right eye and 0.5 in the left eye.

Sub-tenon triamcinolone injections (STTA) were performed in the right eye, and thereafter, intravitreal injection of anti-vascular endothelial growth factor (VEGF) was performed a total of 22 times (aflibercept 12 times; faricimab 10 times). The BCVA fluctuated between 0.2 and 0.9 during this period in the right eye. Pars plana vitrectomy (PPV) was performed on the left eye for the DME and ERM 17 years after the initial visit. This resolved the DME; however, a new PED appeared near the old one 22 years after the initial visit. In addition, subretinal fluid appeared over the PED, and macular neovascularization (MNV) was observed beneath the PED, which had been observed 13 years before (Figures 2C, 2D). His decimal BCVA decreased to 0.4 in the left eye. Since then, IVF has been performed 10 times, and the BCVA has improved to 0.8. Bilateral cataract surgery was performed 17 years after the initial visit. At his most recent visit, 23 years after the initial visit, the BCVA was 0.7 in the right eye and 0.6 in the left eye.

## Discussion

Several epidemiological studies have reported the occurrence of cases of AMD and DM [4,9,10]. According to the Beaver Dam Eye Study, the prevalence of wet AMD was higher in men patients with DM aged ≥75 years than in non-DME patients [4]. The incidence of AMD in DM patients was significantly lower than in the general population [9]. Of 1,739 patients with diabetes mellitus, 183 (10.5%) were diagnosed with AMD, and of 263 DME patients, 12 (4.6%) had dry AMD, and nine (3.4%) had wet AMD [10]. The incidence of AMD was lower in patients with more severe DR [10]. AMD and diabetes share a common pathophysiology, mainly involving inflammatory processes and oxidative stress. However, the relationship between these two has not been definitively determined [1,3-8]. Because there have been no reports showing the progression of both diseases in specific cases, this case provides valuable clinical data to vitreoretinal specialists.

In our case, the OCT macular map that was taken nine years after the initial visit showed RPE irregularities (Figure 2A), and the MNV developed at this site 13 years later (Figure 2D). Because the locations of MNV and DME were different, and the layers of the retina in which they were localized also differed, we believe that the MNV occurred independently of the DME. However, the left eye had undergone PPV for the ERM, and the possibility that this may have contributed to the development of MNV cannot be excluded. Nevertheless, it is not clear which factors were most strongly involved in the development of the MNV. Although both diseases respond to intravitreal anti-VEGF injection therapy in the present case, the optimal drug type may vary depending on the type of AMD or DME. Therefore, when both diseases occur in the same patient or in the same eye, it will be necessary to select the appropriate drug for each case.

## Conclusions

In conclusion, a detailed course of a rare case of AMD in one eye and DME in the other eye is described. The MNV developed near the PED, which had been observed in the OCT image taken 13 years before. Clinicians should be aware that AMD may develop during the course of treatment for DME. This case will draw attention to the possibility of the two diseases coexisting and lead to a deeper understanding of the pathogenesis of both diseases.
